# Migraine Associated with Menstruation An Overlooked Trigger

**DOI:** 10.31729/jnma.6332

**Published:** 2021-06-30

**Authors:** Ashlesha Chaudhary

**Affiliations:** 1 Nepal Medical College, Jorpati, Kathmandu, Nepal

**Keywords:** *medical*, *menstruation disturbances*, *migraine disorders*, *students*

## Abstract

Menstrual migraine is a condition in females, where headaches are linked with menstruation and may be debilitating. Hormonal fluctuations could have a key role in migraine etiopathogenesis, as several women experience that their migraine attacks correlate with their menstrual cycle. Estrogen withdrawal appears to have a significant role in migraine associated with menstrual cycles, despite the fact that its pathophysiology is not well known. The treatment method can also vary from that used to treat nonmenstrual migraines. However, with proper identification and management of the condition, it can be bearable. This article highlights some portions of what is known about migraine, its triggers including the experience of a sufferer and aims to provide readers with a better understanding of migraine in women by understanding these aspects of the condition.

## INTRODUCTION

Menstruation is a natural physiological phase, but even small variations in the normalcy during the cycles threaten the ease and productivity of women's daily routines. Menstrual irregularities, as well as delayed menstruation, are the major concerns primarily due to hormonal fluctuations. Menstrual migraine, also known as catamenial migraine, is a less well-known (10%) problem associated with menstruation.^1^ As compared to attacks outside of the menstrual period, these headaches are often without an aura, last longer, and are accompanied by more serious nausea.

Migraine headaches can occur with or without aura (transient focal neurological symptom). International Headache Society described migraine without aura as recurrent headache attacks that last 4-72 hours, are unilateral, pulsating, mild to severe, exacerbated by regular physical exercise, and associated with nausea, photophobia, and phonophobia.^1^ Menstrually associated migraine (MAM) is a form of migraine that develops without an aura during the perimenstrual period (day 1 of menses±2 days).^2^

## CONTRIBUTING FACTORS

A variety of factors can trigger the onset of a migraine attack, with different mechanisms causing a migraine attack. Even though the condition is often familial, any changes in genes of significant impact sizes have not been yet found by genome-wide association studies.^3^

Besides genetic factors, other implicated factors for the mechanism of migraine attacks are stress, barometric pressure, diet, neuroendocrine function, menstrual cycle, pregnancy, and drugs.^4^

## EXPLORING THE MECHANISM NOT QUITE WELL KNOWN

All migraine sufferers are prone to the effects of hormonal changes. The fluctuating hormones of the regular ovarian cycle are a particular trigger for a subset of women who suffer from menstrual migraines. Estrogen withdrawal before the onset of menses is suggested to be the key according to evidence.^5^ The time duration of exposure to estrogen before its withdrawal is significant in the pathogenesis of this condition as supported by Somerville's work.^6^ A relation between the withdrawal of estrogen and migraine headaches has shown to be evident in users of oral contraceptive pills (OCPs).^7^

Estrogen's role in increasing production, uptake, and elimination of serotonin as well as enhancing the sensitivity of certain serotonin receptors has been suggested.^8^ So, withdrawal of estrogen would decrease the serotonergic tone. This hypothesis is consistent with triptans which are specific serotonin receptor agonists and can avert and treat menstrual migraine.^9-11^

## KNOWN TREATMENT STRATEGIES

In addition to avoiding the triggers, medications are also required to manage migraine headaches. The first line options include Acetaminophen, NSAIDs (Aspirin, Diclofenac, Ibuprofen, and Naproxen) and Triptans (Almotriptan, Eletriptan, Frovatriptan, Sumatriptan, Zolmitriptan).^12^ Frovatriptan, taken two times daily, showed the most and best evidence of all triptans, followed by drugs naratriptan and zolmitriptan for the management of menstrual migraine.^11^ To begin, the patient should seek professional help, understand the situation and follow a proper treatment plan. The medicine should indeed be taken as soon as the headache attack begins.^12^ Proper follow-up and medication modification are essential.

## EXPERIENCES

Missing meals, not having proper sleep, or getting stressed out would cause frequent headaches which would also be triggered at times after drinking a cup of coffee. This pattern was easy to miss for a first-year medical student who associated the headache to be coincidental and normal with the circumstances. During most of the instances, the headache would be unilateral, pulsatile, exacerbated by day-to-day physical activities, mild to severe in intensity, and accompanied by nausea, vomiting, and photophobia.

After several episodes and after being rushed to the emergency multiple times, a pattern became evident and the next step had to be seeking professional help. This led to the diagnosis of a condition named migraine for which analgesic and antiemetic medications were prescribed but what wasn't clear to that student was whether to take the medications after the headache had gotten problematic or as soon as the headache started. After having many episodes of headache, studying the condition, and consulting a couple of doctors, the situation could be managed properly. What worked was the triggers had to be avoided and medications had to be taken as soon as the episode started.

Then, came the recognition of a trigger that was unavoidable for a female, her menses. Headache episodes would occur mostly a day before or during menstruation even after avoiding all other known triggers which led to skipping lectures, not being able to concentrate in classes, and having prolonged resting periods. Further, when the attacks came during exams, the stress would be unbearable.

Gradually, with the help of doctors and after knowing one's body better, recognizing the triggers better, the episodes could be prevented as well as treated better. Besides eliminating known triggers, avoiding vasoactive agents like caffeine, and adhering to consistent eating, sleeping, and exercise routines, what worked was taking a single dose of an analgesic with an antiemetic during the initial phases of the headache.

## WAYS FORWARD

Being an overlooked condition, menstrual migraine needs to be better understood for its appropriate management. Accurate and early diagnosis and proper management of migraine could help students improve their academic performance. Further research could concentrate on the identification of subtypes of migraine and its triggers among medical students. Well-designed strategies to prevent attacks of migraine headaches, as well as an educational program to raise awareness among medical students, are also suggested.
